# HIV risk perception and sexual behavior among HIV-uninfected men and transgender women who have sex with men in sub-Saharan Africa: Findings from the HPTN 075 qualitative sub-study

**DOI:** 10.1371/journal.pgph.0001408

**Published:** 2022-12-27

**Authors:** Yamikani R. Mbilizi Chimwaza, Sufia S. Dadabhai, Alinane L. Nyondo Mipando, Calvin Mbeda, Ravindre Panchia, Jonathan P. Lucas, Wairimu Chege, Erica L. Hamilton, Theodorus G. M. Sandfort

**Affiliations:** 1 Johns Hopkins Research Project–Blantyre CRS, Blantyre, Malawi; 2 Johns Hopkins Bloomberg School of Public Health, Department of Epidemiology, Blantyre, Malawi; 3 Kamuzu University of Health Sciences, Blantyre, Malawi; 4 Kenya Medical Research Institute, Kisumu Clinical Research Site, Kisumu, Kenya; 5 Perinatal HIV Research Unit, University of the Witwatersrand, Soweto, South Africa; 6 Science Facilitation Department, FHI 360, Durham, NC, United States of America; 7 National Institute of Allergy and Infectious Diseases, National Institutes of Health, Bethesda, MD, United States of America; 8 HIV Center for Clinical and Behavioral Studies, New York State Psychiatric Institute and Columbia University, New York, NY, United States of America; University of Toronto, CANADA

## Abstract

There remains a limited understanding of how men who have sex with men (MSM) and transgender women (TGW) in sub-Saharan Africa (SSA) perceive their risk for HIV and how risk influences behavior during sexual interactions. We performed thematic analysis on in-depth interviews from the qualitative sub-study of HPTN 075 in Kenya, Malawi, and South Africa. Using the Integrated Behavioral Model (IBM) constructs, we found that most MSM and TGW perceived themselves to be at risk for HIV, leading them to regularly engage in safer sexual behaviors. Notably, even though these MSM and TGW perceived themselves to be at risk for HIV, some of them reported engaging in transactional sex, sex under the influence of alcohol, and intentional non-use of condoms. This indicates that HIV risk perception was not always associated with safer sexual behaviors or a reduction in risk behaviors. Attitudes (negative attitudes toward condom use), perceived norms (social pressures), and environment constraints (contextual barriers) were related to MSM and TGW not engaging in safe sexual behavior. Hearing the perspectives of MSM and TGW on their sexual behavior continues to be important for the development and implementation of effective prevention policies and interventions. Eliminating structural barriers such as stigma, discrimination, and criminalization of same-sex sexuality is a crucial prerequisite for the success of interventions to promote sexual health among MSM and TGW in SSA.

## Introduction

Globally, men who have sex with men (MSM) and transgender women (TGW) have been disproportionately infected with and affected by HIV since the beginning of the epidemic [1, 2]. In 2019, there were roughly 1.7 million new HIV infections globally; 23% were among gay men and other MSM, and 2% were among TGW [3]. In East and Southern Africa, the region hardest hit by HIV, gay men and other MSM account for 6% of new infections [3]. Overall, one in five (20%) MSM in the region is estimated to live with HIV [3].

In some parts of the world, there are substantial social and legal barriers to achieving adequate coverage (access and uptake) of HIV prevention services for MSM and transgender individuals [4]. Based on the Global Men’s Health and Rights study (GMHR), which included 11% of MSM from sub-Saharan Africa (SSA), a low proportion reported having easy access to free or low-cost condoms, condom-compatible lubricants, HIV testing, HIV treatment, and HIV educational materials targeted at MSM [5]. In many African countries, same-sex sexuality is illegal and punishable by law, contributing to the hostile environment that increases MSM’s vulnerability to stigmatization, discrimination, and violence [4]. For example, same-sex sexuality punishable in Malawi by up to 14 years in prison, although prosecutions were suspended in 2012 [6, 7]. Nevertheless, MSM and TGW still face assault, police brutality, discrimination by health care providers [8], human rights abuses, and sexual assault [9]. Despite the liberal legislation, the lesbian, gay, bisexual, transgender, queer or questioning, intersex, and asexual (LGBTQIA+) communities in South Africa still face strong social stigma and homophobic violence because of traditional and conservative attitudes within the general population [10]. The criminalization of same-sex relationships complicates the development of policies and the implementation of programs [11]. Due to such barriers, MSM and TGW in SSA are left helpless and may develop their own potentially misinformed perceptions and attitudes towards their risk of HIV infection, translating to risky sexual behavior and increasing their susceptibility to HIV infection.

Additionally, they miss opportunities to benefit from effective prevention methods such as condoms and lubricants, pre-exposure prophylaxis (PrEP), and risk reduction counseling. Male same-sex attracted individuals lack the opportunity to discuss high-risk sexual practices with healthcare providers, impeding access to HIV and sexually transmitted infection (STI) prevention and treatment options [12]. National strategic HIV plans from 41 of 45 African countries now include policies for MSM populations [13]; however, there is very little evidence of MSM or TGW utilizing HIV prevention services and programs on the ground in many African countries.

Most research reports a discordance between self-perceived and actual HIV risk among MSM. Several studies in the USA [14, 15], Netherlands [16], and China [17] have reported that over half of MSM perceived that they had low or no risk of contracting HIV, despite their actual behavior. Low levels of HIV risk perception, deficiencies in HIV knowledge, and engaging in high-risk sexual behaviors among TGW are well-documented in non-African populations [18]. Unfortunately, the transgender population in Africa is a hidden and understudied population [19]. Most transgender data is historically not collected separately; instead, it is lumped together with MSM data, which is misleading [19]. There remains a limited understanding of how MSM and TGW in SSA perceive their risk for HIV and how risk influences their behavior during sexual interactions. Literature shows that risk perceptions are an inherent part of the decision-making process and an essential precursor to health-related behaviors for either managing or preventing risks [20]. Therefore, it is essential to understand how MSM and TGW in the African context see their risk of acquiring HIV and integrate this understanding into their sexual behavior.

## Materials and methods

### Setting

In August 2017, one-time, semi-structured in-depth interviews (IDIs) were conducted with 80 participants at all four HIV Prevention Trials Network (HPTN) 075 study sites: Kisumu Clinical Research Site (CRS) in Kisumu, Kenya; Blantyre CRS in Blantyre, Malawi; Soweto HPTN CRS in Soweto, South Africa, and Groote Schuur HIV CRS (Desmond Tutu HIV) in Cape Town, South Africa. Sites were selected considering their research capacity, previous engagement with black MSM to leverage existing relationships with community organizations serving the target population, and the goal for HPTN 075 to include different settings in which same-sex sexuality was either legal (but stigmatized) or illegal and criminalized [21].

### Study design

We analyzed data from the qualitative sub-study of HPTN 075. HPTN 075 was a one-year observational cohort study that enrolled 401 MSM and TGW to evaluate the feasibility of recruiting and retaining MSM in a multi-country prospective cohort in preparation for future HIV prevention studies [21]. HPTN 075 enrolled persons assigned male sex at birth who reported having sex with men. Although no specific recruitment efforts were employed to recruit TGW, TGW were not excluded. The qualitative sub-study explored interest among MSM and TGW in PrEP and systematically addressed the perception and management of the risk of HIV transmission in sexual interactions.

### Population

Participants in the HPTN 075 qualitative sub-study were eligible to participate if they were HIV-uninfected, identified as MSM or TGW, were aged 18 to 44 years, allowed their interview to be digitally recorded, and were able and willing to provide informed consent. Participants were purposively sampled and selected to participate in the HPTN 075 qualitative sub-study. During the week 26 follow-up questionnaire, HPTN 075 participants were asked to indicate their knowledge and interest in PrEP as an HIV prevention intervention. The HPTN Statistical and Data Management Center (SDMC) at SCHARP identified participants’ identification numbers (PIDs) by positive or negative responses to specific questions during the week 26 interview. Based on their level of interest in PrEP, participants were divided into two groups: those with no or limited interest in PrEP and those with a strong interest in PrEP. The PIDs were sent to designated site staff at respective sites to recruit potential participants by PID via their contact information. HPTN 075 research staff at the four sites were educated about male same-sex sexuality and trained to conduct quantitative and qualitative interviews.

### Conceptual framework

The analysis of the data was guided by the Integrated Behavioral Model (IBM), a theory that integrates tenets from the Theory of Reasoned Action and the Theory of Planned Behavior and is a general theory of behavioral predictions assumed to apply to any given situation [22]. The constructs of IBM include Attitude, referring to the degree to which a person has a favorable or unfavorable evaluation of the behavior of interest and a consideration of the outcomes of performing the behavior; Subjective norm, referring to the belief about whether most people approve or disapprove of the behavior and whether peers and people of importance to the person think he or she should engage in the behavior; Perceived Agency, individual’s ability to originate and direct actions for a given purpose, including Perceived Control, the individual’s sense of control over their behavioral conduct, including the person’s sense of whether various environmental factors make it easy or hard to perform the behavior; Self-Efficacy, an individual’s belief in her/his effectiveness to perform a specific behavior; and four additional factors–Knowledge and skills to perform the behavior; Salience of Behavior; Environmental constraints; and Habit [22]. We applied the IBM in this study to determine whether the perception of risk for HIV can help us understand whether or not MSM and TGW in SSA engage in safe sexual behaviors. The study comprehensively explored the views, opinions, and experiences of MSM and TGW that addressed the constructs of the IBM that determine sexual behavior. The attitude constructs assessed the positive and negative feelings MSM and TGW had towards the risk of HIV and existing preventive measures such as condoms or PrEP. The construct of perceived norm described the social pressure some MSM and TGW feel to perform or not perform a specific behavior concerning HIV prevention in sexual interactions. The construct of personal agency, which consists of perceived control and self-efficacy, explains an individual’s confidence levels in their ability to perform a particular behavior (i.e., self-protect against HIV) despite obstacles that stand in their way. The knowledge of HIV transmission and prevention helped assess the accessibility and amount of information in the MSM and TGW communities. Even though the IBM includes other constructs, we identified these main determinants of behavior among the MSM and TGW in this qualitative sub-study. The IBM can also be used for behavioral interventions that help identify which constructs individuals lack and, therefore, need to be addressed to promote behavior change [22, 23]. This study presents the perceptions of risk for HIV infection in the words and experiences of MSM and TGW in SSA, which may help us to understand their sexual behavior.

### Qualitative data collection and analysis

The analysis is based on participant responses to the open-ended questions under the safer sex practices topic of the semi-structured in-depth interview. Other sections covered in the interview included: the background of social networks and oral and injectable PrEP. Interviewers were trained to probe the answers provided by the participants. The interviews were audio-recorded, then transcribed transcripts, and as far as necessary, subsequently translated into English. Transcripts were uploaded and analyzed in Dedoose (version 8.1.9), a software program for qualitative content analysis. The analysis focused on sections of the transcripts related to the discussion of safer sex practices only. A priori codes were derived from the study objective, interview guide, and the primary constructs of the IBM (behavioral attitudes, perceived norms, perceived behavioral control, and knowledge). Two researchers (YM and TS) developed 16 concept-driven codes in an iterative process of drafting, testing, and finalizing each code in the codebook. Subsequently, two coders (YM and KG) independently analyzed an initial sample of excerpts from five interviews. They subsequently compared and discussed their coding results and finalized the codebook once a consensus was reached about the application of the codes. After consistent double coding for a subset of the interviews, independent coding was performed (by YM) for the remaining 75 interview excerpts. The codes were then collated into themes identified by patterns and trends in responses and insights that addressed the research question. This paper’s results and discussion sections indicate quotes from interviews (interviewer and participant responses, or just participants’ responses) in indented italic text and include the participant’s age, sexual identity, and country after each quote.

### Ethical approval

The parent HPTN 075 study and the qualitative sub-study were reviewed and approved by the Institutional Review Boards (IRBs) at the following institutions: Johns Hopkins University School of Public Health; Kenya Medical Research Institute; College of Medicine Research Ethics Committee in Malawi; the University of Cape Town, Faculty of Health Sciences; the Research Ethics Committee (Medical) at the University of the Witwatersrand; and the New York State Psychiatric Institute. Participants provided written consent at three sites and oral consent at one site (Malawi), as directed by the local IRB because a written name and signature could lead to harm if a participant’s identity were discovered.

## Results

### Participant characteristics

A total of 80 participants took part in the in-depth interviews, 20 participants per site. Most participants were under 25 years old (68.8%), had a middle to low level of education (66.2%), were unemployed or students (48.7%), were unmarried (91.0%) and self-reported as currently in a same-sex relationship (79.5%). Sixty (75.0%) of the 80 participants identified as male, while 15 (18.0%) identified as transgender, and 5 (8.3%) did not specify their gender. More than half (55.1%) of all participants reported being sexually attracted to both men and women (compared to 44.9% attracted to men only), and 44.9% reported exclusive same-sex attraction. Being the insertive partner during same-sex anal sex was the preferred option (50.0% vs. 35.9% receptive partner vs. 14.1% no preference). Table 1 displays sociodemographic characteristics.

**Table 1:** Sociodemographic characteristics of MSM and TGW in the HPTN 075 qualitative sub-study, (N = 80).

Characteristics	MSM N = 60 N (%)	Transgender N = 15 N (%)	Gender unspecified N = 5 N (%)	Total N= 80
**Age (years)**				
18–25	39 (65.0)	13 (86.7)	3 (60.0)	55 (68.8)
26–44	21 (35.0)	2 (13.3)	2 (40.0)	25 (32.5)
**Level of education** ^**a**^				
High (some tertiary)	22 (36.7)	3 (21.4)	1 (33.3)	26 (33.8)
Middle (completed secondary)	23 (38.3)	4 (28.6)	1 (33.3)	28 (36.5)
Low (less than secondary)	15 (25.0)	7 (50.0)	1 (33.3)	23 (29.7)
**Employment** ^**a**^				
Full-time/Part-time	23 (38.3)	3 (21.4)	0	26 (33.3)
Self-employed	6 (10.0)	5 (35.7)	1 (33.3)	12 (15.4)
Unemployed/Student	31 (51.6)	6 (42.9)	1 (33.3)	38 (48.7)
Other	0	0	1 (33.3)	2 (2.6)
**Marital status** ^**a**^				
Married	6 (10.0)	0	1 (33.3)	7 (9.0)
Not married	54 (90.0)	15 (100.0)	2 (66.7)	71 (91.0)
**Ongoing same-sex relationship** ^**a**^				
Yes	49 (81.6)	11 (17.8)	2 (66.7)	62 (79.5)
No	11 (18.3)	4 (25.0)	1 (33.3)	16 (20.5)
**Sexual identity** ^**a**^				
Bisexual and other	22 (36.6)	4 (26.7)	1 (33.3)	27 (34.6)
Gay	38 (63.3)	11 (73.3)	2 (66.7)	51 (65.4)
**Sexual attraction** ^**a**^				
Men and women	35 (58.3)	6 (40.0)	2 (66.7)	43 (55.1)
Men only	25 (41.6)	9 (60.0)	1 (33.3)	35 (44.9)
**Preferred sexual role in anal sex** ^**a**^				
Insertive	35 (58.3)	12 (80.0)	2 (66.7)	49 (50.0)
Receptive	16 (26.7)	2 (13.3)	0	18 (35.9)
No preference	9 (15.0)	1 (6.7)	1 (33.3)	11 (14.1)

^a^N does not add up to the total due to missing values.

### Theme one: MSM and TGW had good knowledge of HIV transmission and strategies that can be used to reduce the risk of HIV transmission

To better understand what was known about HIV, how it is transmitted, and how it can be prevented, participants were asked to describe how and in what situations MSM and TGW in their community were most likely to acquire HIV. The following subthemes could be distinguished: correct and misperceptions about HIV transmission, and knowledge about protective measures against HIV acquisition.

### Subtheme one: Correct knowledge

Across all three countries, MSM and TGW were aware of the different modes of HIV transmission (e.g., unprotected sex, multiple sexual partners, and sharing contaminated sharp objects). These participants correctly identified a variety of protective measures, including consistent use of condoms with lubricant; using the right kind of lubricant; maintaining one sexual partner; use of PrEP before sex and post-exposure prophylaxis (PEP) after accidental exposure; knowing your HIV status, and being informed through HIV education. Young (18–25 years) participants were more knowledgeable about HIV and were aware of new and old methods of preventing HIV. MSM and TGW were aware of the greater risk for HIV among uncircumcised males having unprotected sex and the protective benefit that circumcision provides.

*MSM can use safe sex in terms of, maybe, lubricants and condoms because lubricant goes hand in hand with the condom, and it has to be a water-based lubricant, not maybe oil-based or other lubrication… There is PrEP coming in, which is a combination prevention mechanism, and PEP is coming in after you have been exposed…Abstinence, which is very hard, I do not think nowadays if it appears…But at least knowing each other’s status is another way of preventing yourself from HIV. [27 years old, bisexual, Kenya]*

### Sub-theme two: Misperceptions about HIV acquisition

A few MSM and TGW exhibited inaccurate knowledge of HIV transmission. Incorrect perceptions of high-risk exposure to HIV infection were reported by an MSM from Malawi who described how sharing a toothbrush with an HIV-infected person could pass on the virus. A gay man from Kenya believed that having sex with a woman makes someone more susceptible to HIV than having sex with another man.

*It could be during accidents, and you did not know you had blood[y] contact [with another person], [or] you could be in the same house and share a toothbrush. And maybe they are identical toothbrushes, and another person uses it unknowingly; you can pass the virus. [35 years old, bisexual, Malawi]*

*Personally, I think that for you to get that disease easily, it is when you have vaginal sex with a woman. [39 years old, gay, Kenya]*

### Subtheme three: Risk reduction strategies

MSM and TGW were prompted to describe ways they could protect themselves against HIV infection during sex. Most participants said that they used condoms and lubricants during sex, while a few said they tried to abstain when possible.

*First of all, I use condoms. I suggest to my partner that he use condoms at all times. So even if it breaks during intercourse, I tell him to stop, take another one, then we continue with the journey. [20-year-old, transgender, Cape Town]*

There was varying knowledge of oral PrEP across participants in the three countries. However, most participants had heard of PrEP and could mention where they heard about it, how it works, and when to use it. Participants accessed information about PrEP either from health personnel (from hospitals or when participating in HIV studies) or from peers. One participant from Cape Town reported that they were currently using oral PrEP, while another from Soweto said he was eager to access PrEP finally.

*I use a lubricant whenever I am having sex with someone, and I always put obviously a condom [on], and I am also on PrEP. [23 years old, gay, Cape Town]*

*But I just heard that whenever two people are having sex, if one of them took the PrEP medicine before having sex, his body would be very much protected in that if it happened, that during sex, the HIV has entered into the blood of his body, it would not be strong enough to infect the person’s body but would die since the body has strong protection from PrEP. [25 years old, bisexual, Malawi]*

### Theme two: MSM and TGW identified themselves to be at risk for HIV and associated it with risky sexual behavior

This theme illustrates the participants’ understanding of their own or other MSM and TGW’s risk for HIV infection. Answers also covered their sense of control and confidence in protecting themselves (including skills and self-efficacy) during sexual interactions. The two subthemes that emerged were the perception of being at risk for HIV (what the participants perceived as their risk of HIV acquisition) and self-efficacy (how hard or easy it feels to protect themselves against HIV and what makes it hard or easy to do so).

### Subtheme one: A common perception of being at risk for HIV

Across all study sites, most MSM and TGW perceived themselves as at risk of acquiring HIV during their current sexual interactions. Even though participants were aware of risk reduction strategies they could use during sex, some found condoms and lubricant “not fully dependable” or found themselves in situations where alcohol contributed to deliberate and spontaneous acts of risky sexual behavior such as condomless anal sex. One MSM from Malawi mentioned that even though he used condoms, he still felt at risk for HIV, lacking trust in the barrier method.

*Yes, I am at risk. I can say so. Although I try to protect myself by using condoms and lubricants, these are not fully dependable to me, so I still feel I am at risk. [35 years old, bisexual, Malawi]*

*I am [at risk] because of certain things that I do…under the influence of alcohol…and there are certain things that I do just to be naughty. [23 years old, gay, Soweto]*

*I can say for the past last week, I had an incident where I had sex twice without using a condom or anything. It was not something deliberate; it was just in an act where we were both a bit tipsy, and we just wanted to get into action…He had nothing, so we just did it. [20 years old, transgender, Cape Town]*

Other MSM and TGW mentioned that they felt that their risk for HIV was attributed to the fact that they have sex with men and the biological factors involved with penetrative or receptive anal intercourse. Some participants stressed that for MSM, the risk for HIV was more significant than for men who have sex with cisgender women (MSW):

*…well, I’m a bottom. And obviously, the act of having sex with a man, the biological and the anatomical [the] equipment isn’t really designed for that…. So that’s the risk on its own. There could be tearing, there could be scarring, there could be all sorts of things that could happen there, just in the act of having sex, so I think that’s why I see myself being at risk. [28-year-old, transgender, Soweto]*

Another MSM from Kenya attributed his risk for HIV to came from being a receptive partner when engaging in anal sex:

*You know, men who have sex with men are at a higher risk of getting HIV compared to men who have sex with women. [29 years old, gay, Kenya]*

### Subtheme two: Varied levels of self-discipline, self-control, and self-confidence among MSM or TGW determine protective or risky behavior during sexual interactions

The interview prompted MSM and TGW to describe whether it was generally hard or easy to protect themselves against HIV. They were asked to describe what made it hard or easy and which situations were easier or more difficult. Participants described how protection was more effortless when there was mutual understanding and agreement between sexual partners, both for condom use and remaining faithful to each other. Some said it was easier to protect themselves if they avoided sex while under the influence of alcohol or drugs for better self-control and decision-making. Power dynamics in same-sex relationships played a role where self-protection was described as challenging. A gay man from Cape Town described how the insertive sex partner might be controlling whether protection is used during sex in some MSM relationships, which leaves the less powerful partner at possible risk of acquiring HIV. Even if the receptive sex partner was aware of the risk for HIV, if they did not or could not negotiate the use of protection and had to ’go with the flow,’ they were put at risk of HIV. However, there were some MSM who described how they were independent and controlled their protection during sex. Knowing one’s HIV status and making every effort to maintain a negative status made one participant report how he avoids having sex with someone who could infect him. Being aware of the consequences of unprotected sex made most participants feel confident and capable of using protection and avoiding contracting HIV consistently. One man from Soweto boasted that he had mastered his way of staying HIV-free, unlike his other friends.

*They let their dominant partner take control, and they do not question any situation that comes with that. Even if it is risky to themselves, they just say go with the flow, which is how it makes them very much easier to be infected. [26 years old, gay, Cape Town]*

*I find it easy for myself. I’m very wise when it comes to sex; they call me a master. [22 years old, gay, Soweto]*

### Theme three: Fear of acquiring HIV drove most MSM and TGW to curtail risky sexual behavior

All 80 MSM and TGW were HIV uninfected at the time of the interview. The attitudes coded in this theme reflect their feelings and emotions toward the potential risk of acquiring HIV in their current sexual interactions. The only feeling that emerged was the fear of the acquisition of HIV and possible implications, such as discrimination because of living with HIV. When self-reported, all participants were fearful of the possibility of acquiring HIV. Fear was the primary motivator for most MSM and TGW to use protection to prevent HIV acquisition. However, for some, the same fear was a hindrance to getting HIV tested. Some participants were conflicted by worrying about not knowing their HIV status and the apprehension and fear of getting a positive HIV test result. As a result, they would rather stay away from accessing HIV services at health facilities.

*I see that I am at risk, and I try my best to protect myself from the HIV by using condoms. [21-year-old, bisexual, Malawi]*

### Theme four: Fear of HIV discrimination was a barrier to accessing HIV testing and counseling

Some participants would not seek HIV testing services due to fear of discrimination of being found to be HIV-positive. A Kenyan transgender, for example, described the issue of fear of an unknown HIV status, and when you are too terrified to find out your HIV status for fear of receiving a positive test.

*The fear of going to the clinic is that same fear of not knowing what is happening to you and already you are suspicious that maybe you could be found with a disease, you can be found with [[HIV]AIDS because the disease is common …., and therefore, you are already fearful, and you do not want to go to the clinic. That is what is making it difficult sometimes for some people to go to the clinic for information. [24 years old, transgender, Kenya]*

### Theme five: Some mistrust in condoms for protection among MSM and TGW who prioritized pleasure over protection

This theme identified opinions and feelings towards HIV protective measures and their use during sex, regardless of whether participants were currently using them or not. We identified that the negative attitude towards condoms stemmed from various factors such as the reduced feeling of pleasure, barrier effectiveness (possibility of breakage), and lack of trust in their effectiveness.

**Subtheme one: Negative attitudes towards protective measures against HIV.** MSM and TGW reported negative attitudes towards protective measures, specifically the male condom. Some participants reported how they did not like how a condom feels and how it hinders the real pleasure of sex. A gay man from Malawi mentioned how he is not happy using condoms and prefers not to use one. Another man mentioned how sometimes he even felt the urge to remove the condom during sex to ’feel great.’ Other MSM and TGW expressed their doubts by emphasizing how condoms are "supposed" to be used most of the time and "supposed’’ to be treated as the best prevention methods, yet they were not fully convinced of their effectiveness.

*We are not happy to use condoms when having sex. It is difficult since I do not like using condoms most of the time. [23 years old, gay, Malawi]*

*The condom will not bring out the real pleasure" [35 years old, bisexual, Malawi]*

### Theme six: Different social settings in MSM and TGW communities determine if protection can be used

This theme captured the nature of sexual interactions and practices among the MSM and TGW that either put them at increased risk or reduced risk for acquiring HIV. This may be a direct consequence of individual perceptions of risk or described as the normative in their community. MSM and TGW There mentioned many examples of risky sexual. Where alcohol was involved, one man recognized the poor decisions he made and how he became fearless of the risk of possible exposure to HIV and other diseases during unprotected sex. A man from Kenya described how money’s influence in exchange for sex (transactional sex) made other men not question a partner’s HIV status or disregard using protection. Another participant mentioned that group sex was a common practice among MSM and TGW, and sometimes it involved having sex with multiple strangers.

*Most commonly, when I am drunk, it becomes very difficult for me, I make poor decisions, and thus, one is not scared of having unprotected sex with another person. [35 years old, bisexual, Malawi]*

*As long as you have been given money, you will go with him without protection and so there are people who forget to protect themselves.…, now money makes you misbehave and at the same time forget that we should protect our lives*.

*[24 years old, transgender, Kenya]*

*“MSM like group sex and by the time someone does group sex, you don’t know all [these] people you are having sex with person A, person B, person C, and [there] are 4 of you in the same room and you are having sex.” [27 years old, bisexual, Kenya]*

## Discussion

Using the IBM constructs, we found that most MSM and TGW perceived themselves to be at risk for HIV, leading them to regularly engage in safer sexual behavior. The perception of risk was associated with their knowledge of HIV transmission routes, their attitude (fear) toward the possibility of contracting HIV, and their awareness of varying levels of perceived control and ability (self-efficacy) to protect themselves during sexual encounters.

Most participants were MSM, and our findings are consistent with previous and recent studies indicating that MSM who engage in risky sexual behavior are likely to perceive themselves to be at risk or at high risk for HIV infection [17]. Less is known about the HIV risk perception and sexual of transgender women in Africa. Notably, even though these MSM and TGW perceived themselves to be at risk for HIV, some of them reported engaging in transactional sex, sex under the influence of alcohol, and intentional non-use of condoms. This indicates that HIV risk perception was not always associated with safer sexual behavior or a reduction in risk behavior. There are numerous plausible explanations for why the perception of HIV acquisition risk does not predict or prompt protective (such as avoidance or using protective measures). Research on risk perception demonstrates that individuals frequently engage in risky conduct despite being aware of the dangers and adverse consequences [24]. The perceived benefits of action (pleasure over protection using a condom), sensitivity to reward (money expected after unprotected sex), risk propensity (group sex is common among MSM), feelings of invulnerability (complacency after knowing the partner’s HIV status), social context (stigmatization, discrimination, and criminalization of same-sex sexuality), or levels of impulsivity (spontaneous and casual sex with strangers) [24, 25] were some of the factors we found as probable reasons for this study’s contradicting risk perception and risk-taking. Robles et al [25] implied that as impulsivity increases, risk avoidance decreases regardless of perceived risk [25]. In this study, some MSM reported risky sexual that can be considered impulsive-like. Consider, for instance, the young transgender woman from Cape Town who reported unprotected sex on two occasions during the previous week, claiming that the act was unintended, but because alcohol was involved and there was no access to condoms, they "just did it." This suggests that impulsivity may play a role in individual differences between risk perception and actual risk-taking [25]. As we stated in the introduction, same-sex sexuality is stigmatized and discriminated against in the social context of sub-Saharan Africa. The societal pressures that restrict the freedoms of same-sex relationships and compel secrecy regarding sexual interactions may exacerbate risk [26–29]. There is pressure on same-sex attracted men to engage in sexual activity when opportunities arise, regardless of whether they have condoms and lubricant available [29].

The HPTN 075 qualitative sub-study took place in 2017, at a time when programs to reach key populations (which include MSM and transgender people) with HIV prevention, testing, and treatment services were ongoing in South Africa [30] and Kenya and launched in Malawi [4, 31]. Since HPTN 075 was concluded, the HIV service landscape for MSM and TGW has undergone significant change. For instance, the government of Malawi has shown strong political will and commitment to scale up effective and efficient tailored high-impact interventions for MSM and TGW populations in the current 2020–2025 National Strategic Plan for HIV and AIDS for Malawi [32]. The South African government has had more years addressing HIV-related health issues for MSM and TGW, and in addition to comprehensive HIV services, sites now provide STI screening and treatment, psychosocial support, and counseling, including hormone replacement therapy for transgender people, and more. By September 2021, South African data showed that MSM was the largest key population group initiating PrEP, with steep increases since the start of the rollout [33]. Kenya has made progress in addressing the social and legal barriers that MSM and TGW face due to stigma and discrimination, as well as human rights violations. Hundreds of key population paralegals have been trained to represent LGBTQIA+ communities in response to human rights violations, including crisis management and reporting systems to report incidents and request legal counsel [34].

Despite these policy changes, our study findings continue to be relevant for the development of MSM and TGW-inclusive policies and the development of MSM and TGW-specific al change interventions. The integrative model’s approach to intervention design is predicated on the premise that effective interventions are tailored to the needs of an individual or population [35, 36]. The theory conceptualizes these needs as the variables (attitudes, perceived norms, self-efficacy, environment restrictions, knowledge, and salience) that determine the behavior of the population an intervention seeks to influence [35]. Once these factors for the behavior in the population have been identified, an intervention can be developed to target these variables [35–37]. The rationale behind this approach is that the greater our understanding of the variables that influence health behavior in the target population, the more effectively we can develop interventions to alter the behavior [37]. Intervention design anchored in the IBM should be informed by research [35]. In our study, attitudes proved to be an important determinant of engaging in protective or risky sexual behavior and were related to the intention to perform the behavior. Knowledge (of HIV transmission routes), attitudes (fear of acquiring HIV), and self-efficacy (to use protective measures) enabled most MSM and TGW to curtail risky sexual behavior and practice safe sex. Attitudes (negative attitudes toward condom use), perceived norms (social pressures), and environment constraints (contextual barriers) were related to MSM and TGW not using protection. In this regard, an ideal intervention for MSM and TGW in sub-Saharan Africa must have two routes to behavior change: one component to change attitudes that are negatively associated with the intention to reduce risky sexual behavior, and another component to reinforce attitudes that support safe sexual behavior. However, the success of such interventions will be contingent on social and structural changes. Experiences of stigma, discrimination, and human rights violations have been demonstrated to act as structural barriers to MSM and TGW’s willingness to participate in HIV prevention interventions, particularly those situated at healthcare facilities [38]. In addition, addressing mental health issues and supporting substance use is crucial as these psychosocial factors seriously hamper the efforts of MSM and TGW to protect themselves against HIV infections [39–44]. Consequently, eliminating stigma, discrimination, and criminalization of same-sex sexuality is a critical condition for successful interventions to promote sexual health among MSM and TGW in SSA.

A limitation of the current study is that data were not analyzed separately for MSM and TGW, while it is likely that the identified factors affect their sexual practices differently. Although the study was done several years ago, the results are still relevant due to the paucity of information about TGW in SSA. Studies that focus specifically on TGW in SSA are urgently needed. In addition, future research should include a mixed-methods approach and longitudinal analysis of the relationship between risk perception and behavior among MSM and TGW.

## Conclusions

In the HPTN 075 qualitative sub-study among MSM and TGW in SSA, the use of the IBM as a framework provided an in-depth understanding of the attitudes, perceived control, self-efficacy, the salience of behavior, and environmental constraints that contribute to conflicts in risk perception and behavior among MSM and TGW. Due to multiple factors, the self-perceived risk for HIV infection did not always correspond with sexual behavior. Hearing the perspectives of MSM and TGW on their sexual behavior continues to be important for the development and implementation of effective prevention policies and interventions. Eliminating structural barriers such as stigma, discrimination, and criminalization of same-sex sexuality is a crucial prerequisite for the success of interventions to promote sexual health among MSM and TGW in SSA.

## Supporting information

S1 Text A qualitative HPTN 075 substudy: In-depth interview guide. (PDF)
