# Supplementary material for: HIV risk perception and sexual behavior among HIV-uninfected men and transgender women who have sex with men in sub-Saharan Africa: Findings from the HPTN 075 qualitative sub-study
Source: PLOS Glob Public Health. 2022 Dec 27;2(12):e0001408. doi: 10.1371/journal.pgph.0001408 (PMC10021518; doi:10.1371/journal.pgph.0001408)
Supplement: S1 Text — (PDF) [file pgph.0001408.s001.pdf]

A QUALITATIVE HPTN 075 SUBSTUDY: IN-DEPTH INTERVIEW GUIDE

	**Background**
1	I first want to discuss some words that we will be using in this interview, to make sure that we understand them in the same way.<br><br>There are several different words to describe men who have sex with men, such as gay, bisexual. What word do you use to describe your sexuality?<br>- What do you think of when you hear the phrase, "Men who have sex with men?" What type of person fits into that category? Do you consider yourself to be part of that group?
2	Can you tell me something about the people that you spend your free time with?<br>- Are they friends or family?<br>- Are they men or women?<br>- How many gay men/MSM do you have among your friends?<br>- Do people who you spend your free time with know that you have sex with men?
	**Safer sex practices**
3	What kinds of things can MSM do to protect themselves against HIV infection?<br>- Do you know other ways in which men who are HIV negative can protect themselves?
4	In general, do you think that it is hard or easy for MSM to protect themselves against HIV infection?<br>- What makes it hard?<br>- What makes it easy?<br>- Are there specific situations in which it is easier or more difficult?
5	How do you think men in your community are most likely to become infected with HIV (for instance in what kind of situations, with what kind of partners)?
6	How do you think of HIV prevention, do you see yourself as at risk?
7	Is there anything you do to protect yourself against HIV infection when you have sex?<br>- Are there other ways in which you protect yourself?
8	How is it for you to protect yourself against HIV infection: would you say that that is hard or easy?<br>- What makes it hard?<br>- What makes it easy?<br>- Are there specific situations in which protecting yourself is particularly difficult?

	Oral PrEP
9	We now have another way of protecting oneself against HIV infection. It is called PrEP, or pre-exposure prophylaxis. Have you ever heard about PrEP?<br>- Where have you heard about PrEP, or from whom?<br>- What have you heard about PrEP?<br>- How would you describe what PrEP is?<br>- How do you understand that PrEP protects people?<br>- Do you know anybody who has taken PrEP?
10	To make sure that we are talking about the same topic, let me give you a description of what PrEP is.<br><br>PrEP stands for pre-exposure prophylaxis. It is a pill that you take once a day and that can keep you HIV negative. If you take it every day, PrEP can lower the risk of getting HIV by 92% - 99%. This medication is also used by people who are HIV positive to fight off the virus.<br><br>To be clear, PrEP differs from PEP. PEP stands for post-exposure prophylaxis; PEP is taking HIV medication to prevent infection after someone might have been exposed to HIV.<br><br>PrEP has been shown to be very safe. As with any medication there may be minor side effects, though. Some people get an upset stomach when they first start taking it. In order to get PrEP, you have to see a doctor every 3 months, to get an HIV test, to refill your prescription, and to follow-up.<br>In the United States, PrEP is approved by the Food and Drug Administration. In our country [FILL IN CURRENT STATUS OF APPROVAL].<br>- Do you have any questions about PrEP?
11	How do you think the MSM you know would respond to learning about PrEP, what would they think about it?<br>- Would the MSM that you know be interested in using PrEP?<br>- Why do you think that MSM would be interested in PrEP?<br>- Could you think of any reasons why MSM would <u>not</u> be interested in using PrEP?<br>- Would PrEP be better for some types of MSM than for others? Can you explain?<br>- What kind of men do you think would be interested in using PrEP?<br>- What would you think of someone who uses PrEP to protect himself?
12	How would you personally feel about using PrEP?<br>- Would you yourself be interested in using PrEP?<br>- What do you see as the advantages of using PrEP?

	- What do you see as the disadvantages of using PrEP?<br>- If you compare PrEP with the other ways of protecting oneself against HIV infection that we have talked about before, which one would you prefer most? Can you explain?
13	How do you think people might respond if they found out you were using PrEP? Think of friends, family, and sexual partners.<br>- Can you think of reasons people might disapprove of it?<br>- Who would disapprove of it?
14	Can you think of reasons that people would approve/support you using PrEP?<br>- Who would approve of it and support you?
16	As we mentioned before, the medication used for PrEP is the same medication that HIV-positive individuals take. How would you feel if somebody saw your PrEP medication and thought that you were HIV-positive?<br>- Can you talk about whether that might concern you?
17	As of now, taking PrEP involves taking a pill every day and having your blood drawn every 3 months to get tested for HIV and STI.<br>- What are some of the challenges people might face to staying on PrEP?<br>- What are factors that might help people stay on PrEP?
18	How would you feel about seeing a doctor every 3 months to get tested for HIV and to refill your prescription?<br>- How might that impact your interest in PrEP?
19	Imagine that it would be possible for you to start taking PrEP tomorrow. How would you respond? What might your decision be?<br>- How likely is it that you would start using it, would you say very likely or very unlikely?<br>- Can you explain?
	**Injectable PrEP**
20	One of the disadvantages of PrEP as a pill, is that you would have to take it every day. Otherwise it is not effective. For that reason, researchers are exploring other ways to administer PrEP. One way is by injecting PrEP with a needle into someone's buttocks. We call that "injectable PrEP." Such "shots" are long acting; that means that they stay in your body for a long time.<br><br>There are now studies going on to test whether such an injection is safe and whether it will work to protect people from getting HIV.
21	Imagine that injectable PrEP is safe and it protects people from getting HIV just as well as

	oral PrEP does. People who decide to use it would probably have to get an injection every 2 months and they also have to have blood drawn to test for HIV and STI each time.<br><br>What do you think: would the MSM that you know be interested in injectable PrEP?<br>- Why do you think that MSM would be interested in injectable PrEP?<br>- Are there reasons why they might not be interested?<br>- What do you think that MSM would prefer more: taking a pill every day or getting an injection in their buttocks once every 2 months?
22	What about for yourself: If you were to take PrEP, what would you think about taking a pill every day compared getting an injection in your buttocks once every 2 months?<br>- Can you explain?<br>- What might be some of the advantages of taking a pill every day compared to an injection?<br>- What might be some of the advantages of having an injection every 2 compared to taking a pill every day?
23	How do you feel in general about getting injections, for instance for vaccines?<br>- What kind of injections have you ever received?<br>- How did you feel about these injections?
24	PrEP will be injected with a needle that is relatively thick, like this one [SHOW NEEDLE]. From studies we have learned that injections with PrEP can be painful and that the pain can last for several days. Some people had a sore butt for several days and that made it hard for them to do their work. Other people have said that they experienced irritation, skin redness, bumps, swelling, itching, and bruising where they got the shot.<br>- How would you feel about this?<br>- How might this impact your opinion about injectable PrEP?
25	Injectable PrEP also has other potential side effects such as headaches, diarrhea, fatigue, muscle aches, nausea, fever and dizziness.<br>- How would you feel about such side effects?<br>- How might this impact your opinion about injectable PrEP?
26	One of the potential advantages of injectable PrEP is that it is long acting. That means that a shot stays in your body for a long time, up to one year. But that also means that If you develop side effects after the shot, there will be no way to remove the drug from your body.<br>- How would you feel about this?<br>- How might this impact your opinion about injectable PrEP?
	**Participation in injectable PrEP efficacy studies**

27	As I told you, there are now studies going on to test whether such a PrEP injection is safe and whether it will work to protect people from getting HIV.<br><br>I would like to know whether you, in principle, would be interested in participating in such a study.<br>What are your initials thoughts about this?<br>- Could you explain why you would (not) be interested in participating in such a study?<br>- What would it depend upon?<br><br>IF DEFINITELY NOT INTERESTED: END INTERVIEW
28	As part of these studies, you would also be expected to participate in several medical exams. Your blood will be drawn at each visit. Study staff will also perform a swab of your rectum and collect urine.<br>- How would you feel about this?<br>- How might this impact your interest in participating in such a study?
29	These kinds of studies last a long time. One of the current studies will last for more than four years and study participants have to come to the clinic more than 50 times.<br>- How would you feel about this?<br>- Would it be possible for you to come so often to the same place over such a long period of time?<br>- How might this impact your interest in participating in such a study?
30	People who participate in such a study are placed in one of two groups. The first group gets the injection with the drug in it. The other group will get something called a “placebo” that does not contain the real drug. Placebos look and feel like the real drug, but they do not contain any of the real drug or any other medicines. Comparing the two groups allows researchers to see whether a drug works.<br><br>The group you would be assigned to would be chosen randomly. It is like flipping a coin. You cannot choose which group you are assigned to. You will also not know what group you are in. Until the end of the study, not you or the study researchers, doctors or nurses will know which group you were in.<br>- How would you feel about this?<br>- How might this impact your interest in participating in in such a study?<br>- Imagine that you were to participate in such a study: how likely do you think it is that you would be assigned the group that gets injected with the real drug?<br>- Would you say it is very likely, likely, unlikely, or very unlikely, or would the chances be even? Can you explain your answer?
31	One of the reasons for doing a study like this is to figure out if the drug really protects against HIV infection. That means that if you are in the group that gets the injection with

	the real drug, it is not clear that it protects you against HIV infection.<br>- How would you feel about this?<br>- How might this impact your interest in participating in such a study?
	**End**
32	Is there anything that we have not discussed yet that you think might be relevant?
33	Would you have any questions for me?
